# Supplementary material for: Causal Relationship between Aging and Anorexia Nervosa: A White-Matter-Microstructure-Mediated Mendelian Randomization Analysis
Source: Biomedicines. 2024 Aug 16;12(8):1874. doi: 10.3390/biomedicines12081874 (PMC11351342; doi:10.3390/biomedicines12081874)
Supplement: Supplementary file 1 [file biomedicines-12-01874-s001.zip › Supplementary Figure/Figure S4/Singe SNP plot of MR analysis for TL to ubm-b-1721.pdf]

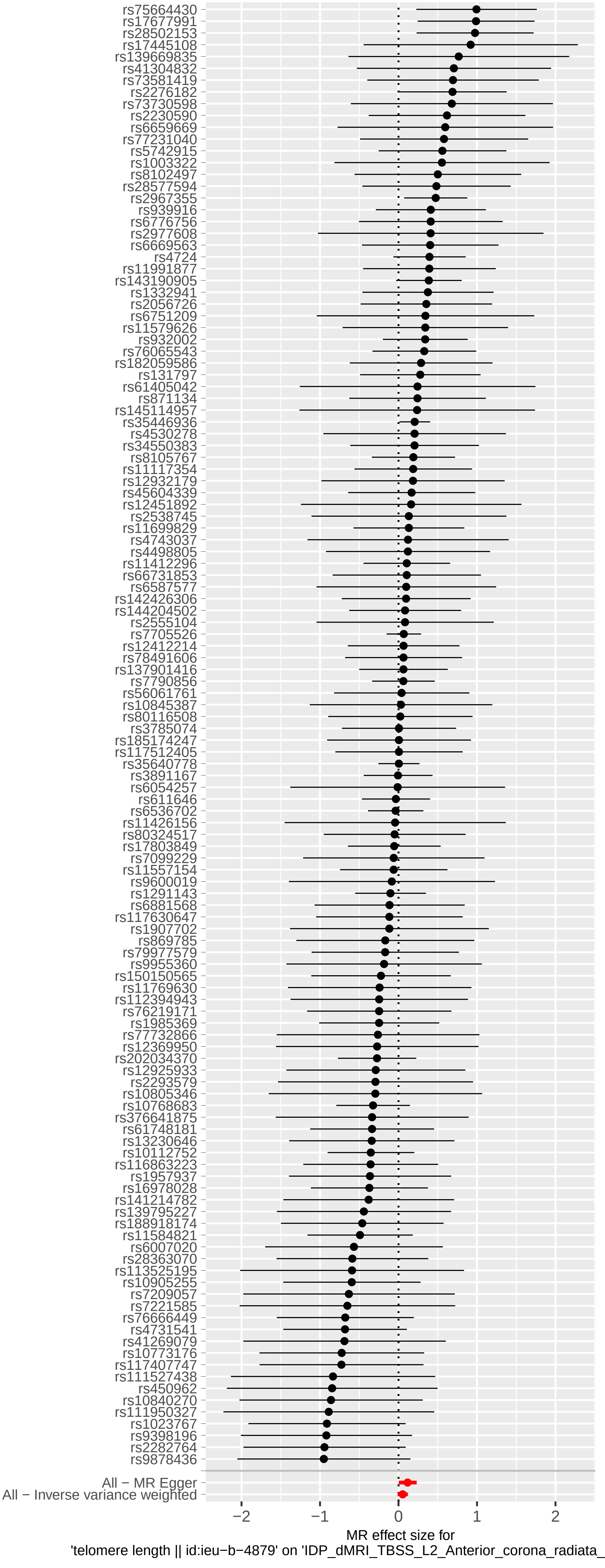
rs75664430
rs17677991
rs28502153
rs17445108
rs139669835
rs41304832
rs73581419
rs2276182
rs73730598
rs2230590
rs6659669
rs77231040
rs5742915
rs1003322
rs8102497
rs28577594
rs2967355
rs939916
rs6776756
rs2977608
rs6669563
rs4724
rs11991877
rs143190905
rs1332941
rs2056726
rs6751209
rs11579626
rs932002
rs76065543
rs182059586
rs131797
rs61405042
rs871134
rs145114957
rs35446936
rs4530278
rs34550383
rs8105767
rs11117354
rs12932179
rs45604339
rs12451892
rs2538745
rs11699829
rs4743037
rs4498805
rs11412296
rs66731853
rs6587577
rs142426306
rs144204502
rs2555104
rs7705526
rs12412214
rs78491606
rs137901416
rs7790856
rs56061761
rs10845387
rs80116508
rs3785074
rs185174247
rs117512405
rs35640778
rs3891167
rs6054257
rs611646
rs6536702
rs11426156
rs80324517
rs17803849
rs7099229
rs11557154
rs9600019
rs1291143
rs6881568
rs117630647
rs1907702
rs869785
rs79977579
rs9955360
rs150150565
rs11769630
rs112394943
rs76219171
rs1985369
rs77732866
rs12369950
rs202034370
rs12925933
rs2293579
rs10805346
rs10768683
rs376641875
rs61748181
rs13230646
rs10112752
rs116863223
rs1957937
rs16978028
rs141214782
rs139795227
rs188918174
rs11584821
rs6007020
rs28363070
rs113525195
rs10905255
rs7209057
rs7221585
rs76666449
rs4731541
rs41269079
rs10773176
rs117407747
rs111527438
rs450962
rs10840270
rs111950327
rs1023767
rs9398196
rs2282764
rs9878436
All − MR Egger
All − Inverse variance weighted
−2
−1
0
1
2
MR effect size for
'telomere length || id:ieu−b−4879' on 'IDP_dMRI_TBSS_L2_Anterior_corona_radiata_
